# Posttraumatic growth and death anxiety in caregivers of cancer patients: PHOENIX study

**DOI:** 10.3906/sag-2001-228

**Published:** 2020-08-26

**Authors:** Ali ALKAN, Elif Berna KÖKSOY, Ebru KARCI, Aslı ALKAN, Eduardo BRUERA, Filiz ÇAY ŞENLER

**Affiliations:** 1 Department of Medical Oncology, Faculty of Medicine, Muğla Sıtkı Koçman University, Muğla Turkey; 2 Department of Medical Oncology, Faculty of Medicine, Ankara University, Ankara Turkey; 3 Department of Medical Oncology, Bağcılar Education and Research Hospital, İstanbul Turkey; 4 Department of Anesthesiology and Reanimation, Faculty of Medicine, Muğla Sıtkı Koçman University, Muğla Turkey; 5 Department of Palliative, Rehabilitation, and Integrative Medicine; the University of Texas MD Anderson Cancer Center, Houston, Texas USA

**Keywords:** Posttraumatic growth, death anxiety, caregivers, relatives, cancer

## Abstract

**Background/aim:**

Posttraumatic growth (PTG) is defined as positive psychological changes following a challenging or traumatic life event. The purpose of this study is to define the predictors of PTG and death anxiety (DAN) in caregivers of cancer patients and evaluate the impact of DAN on PTG.

**Materials and methods:**

The caregivers of cancer patients were evaluated using structured questionnaires, including a validated PTG scale and Templer death anxiety scale.

**Results:**

In 3 different cancer centers, 426 participants were evaluated. In multivariate analysis of factors associated with PTG, a high DAN score was the only parameter associated with high PTG scores [OR: 1.6, CI (95%) 1.02–2.5, P = 0.03]. In multivariate analysis of factors associated with DAN, female sex was the only risk factor for high DAN scores [OR: 1.6, CI (95%) 1.1–2.8, P = 0.049]. There was a positive correlation between PTG and DAN scores (r = 0.15, P = 0.001). Higher DAN scores were associated with positive impacts on self-perception (37.0 versus 35.0, P = 0.02), philosophy of life (16.0 versus 13.0, P = 0.035), and changes in relationship (16.0 versus 14.0, P = 0.01)

**Conclusions:**

This is the first report regarding the association between DAN and PTG. We found a positive impact of death anxiety on psychological changes in caregivers of cancer patients.

## 1. Introduction 

Caregiving a loved one diagnosed with cancer involves providing important emotional, practical, and physical care. However, it is a complex and sometimes overwhelming task. The caregivers of cancer patients (CCPs) are exposed to psychosocial and physical problems, e.g., psychological distress, a decrease in quality of life, and lack of satisfaction in relationships. However, people facing highly stressful life events such as cancer may experience both negative and positive outcomes [1]. 

Posttraumatic growth (PTG) is defined as positive psychological changes that occur following a meaningfully challenging or traumatic life event [2]. After a traumatic event, an individual’s assumptions about the world, themselves, and others are damaged. This change causes a reevaluation and rebuilding of belief systems [3]. A diagnosis of cancer and its consequences may become a series of traumas for CCPs. However, it is hypothesized that CCPs may experience positive changes, e.g., closer relationships with others, a greater appreciation of life, clarification of life priorities, increased faith, and more empathy for others [4]. The factors influencing PTG in CCPs are social support, quality of the spousal relationship, spouse’s PTG, younger age, intrusive thoughts, and marital satisfaction; there is an increase in PTG with shorter diagnosis periods [5,6].

Death anxiety (DAN) is a group of psychological reactions originating from the idea that the self does not exist [7]. The presence of an incurable disease and conscious awareness of mortality can promote DAN [8]. Death anxiety causes a decrease in quality of life, both in patients and CCPs [9,10].

In the literature, PTG has been studied from different perspectives, and there are different models to explain its origin. The Janus-face model explains PTG as a defensive reaction against trauma [11]. In addition, to avoid its deleterious effects, the survivors of trauma try to change losses into benefits. We hypothesized that fear of death, and the related DAN, could motivate individuals to mobilize against the trauma. There is limited data about the positive impacts of DAN on cancer patients and CCPs. Gunst et al. demonstrated a positive correlation between fear of death and PTG in adolescent cancer patients [12]. Luszczynska et al. evaluated the effect of mortality reminders on PTG in breast cancer survivors [3]. They concluded that women exposed to mortality reminders reported lower PTG. In light of limited data about DAN and PTG, studying CCPs who are continuously exposed to psychological trauma and its consequences may provide valuable data for the field of PTG. This study aims to define the predictors of PTG and DAN in CCPs and to evaluate the impact of DAN on PTG.

## 2. Materials and methods 

The study was designed as a multicenter survey and was conducted in 3 cancer centers in Turkey. An institutional ethics committee approved the study protocol, and the study was carried out following the ethical standards of the 1964 Declaration of Helsinki. All the participants signed informed consent. 

The caregivers of cancer patients admitted to outpatient clinics were evaluated. Individuals who were ≥18 years of age were included. The study was held in outpatient clinics that cared for patients over 16 years of age with all types of cancers at any stage. To evaluate the effects of the disease, we included the relatives of patients in remission, patients undergoing adjuvant or palliative therapy, and in those in palliative care. Those with a history of cancer or neuropsychiatric illness that impeded participation in the survey were excluded. During statistical analysis the ages of participants were grouped according to the median age of 40. Income parameters were grouped according to the average wage in Turkey (i.e. 2000 TL) and divided into low or high income. In addition, the length of follow-up was divided into long or short according to the median follow-up time (6 months), and educational status was analyzed as illiterate/literate versus additional education.

The caregivers of cancer patients were evaluated using structured questionnaires; illiterate individuals were evaluated using face-to-face interviews. The questionnaires collected demographic data, information on sociocultural background (presence of siblings, monthly household income, etc.), comorbidities, educational status, job status, and history of psychiatric admissions. In addition to evaluating the effects of patient characteristics on caregiver parameters, information regarding patient age, primary diagnosis, time to follow-up, and disease status was obtained from medical reports. The attending physician recorded the relation of the participant. Participants were asked about their attitude towards screening tests after the cancer diagnosis of their loved ones. Additionally, a question asking them to score the impact of the diagnosis on daily life was added; participants were asked to score according to the Likert scale (very low, low, moderate, high, and very high). Scores of high and very high were analyzed as a high level of impact. To assess DAN and PTG, the validated PTG scale and Templer DAN scale were used [13]. The validity and reliability of the Turkish version have been tested by Senol et al. [14] and Akça et al. [15]. These studies demonstrated test–retest reliability of r = 0.86 (P < 0.001) and 0.79, respectively. The death anxiety scale consists of 15 items, self-report, and a 2 point Likert instrument. The statements are assessed as wrong and right and scored as 0 and 1, respectively. The sum of the 15 items results in a score ranging from 0–15. Scores ≥7 are defined as high DAN. Assessment of PTG was performed by PTG inventory [2]. The psychometric properties of the inventory have been tested in the Turkish population by Dirik et al. [16] and Kağan et al. [17]. Both analyses showed the validity and reliability of the test in Turkish individuals. The instrument includes 21 items rated on a 6 point Likert scale (0–5). The sum of the 21 items results in a score ranging 0–105. Higher scores mean positive psychological changes due to adverse life events. There are subscales of the inventory to evaluate growth in self-perception, philosophy of life, and changes in relationships. In the current analysis, the median score of the PTG scale (70.0) was used to group PTG into high and low.

### 2.1. Statistical analysis 

Baseline characteristics of the patient group were described by using frequencies and proportions for dichotomous and categorical variables. Univariate analysis of the predictors of high DAN and PTG scores was performed by chi-square or Fisher exact tests. Parameters with a P-value less than 0.10 were further analyzed in multivariate analysis. Using a logistic regression model, several parameters were further tested for PTG in multivariate analysis. These included being a spouse, being over 40 years of age, being married, female sex, siblings, and high DAN scores. For DAN, caring for elderly patients, female sex, siblings, low income, not working, the presence of chronic disease, history of psychiatric admission, and high PTG scores were analyzed. The correlation between PTG and DAN was tested using the Pearson correlation coefficient. All analyses were performed using SPSS 17.0 for Windows (SPSS Inc., Chicago, IL, USA), and P-values below 0.05 were considered statistically significant.

## 3. Results 

Between August 2017 and April 2018, 426 participants were evaluated in 3 different cancer centers. The median age was 40.5 years (17–70), and 50.2% were female (Table 1); 58.9% were 1st-degree relatives, and 61.7% were living in the same house. One hundred ninety-five patients (45.8%) were more than 65 years of age, and most diagnoses were gastrointestinal (29.3%) and breast (23.9%) cancers (Table 2). Among the patients, 240 (56.3%) were under palliative chemotherapy or radiotherapy. Three hundred sixty-one participants (84.7%) declared that the diagnosis had a high level of impact on their daily lives. In addition, 26.1% of participants had a screening for malignancy after the diagnosis of their relative. 

**Table 1:** Characteristics of participants.

Characteristics	n(%)
Age (median/range) More than 40	40.5(17–70) 213(50.0)
Female	214(50.2)
Marital status Married Single/divorced	322(75.6) 104(24.4)
Children present	316(74.2)
Live in City center Town/village	287(67.4) 139(32.6)
Live in Self contained house Apartment	191(44.8) 235(55.2)
Monthly income <1000 TL 1000–2000 TL 2000–4000 TL >4000 TL Low income (<2000 TL)	77(18.1) 146(34.3) 141(33.1) 62(14.6) 223(52.3)
Education Illiterate/literate More	33(7.7) 393(92.3)
Job Retired Working Not working	50(11.7) 171(40.1) 205(48.2)
Chronic disease present	137(32.2)
History of psychiatry admission	82(19.2)
Degree of relationship Spouse 1st degree 2nd degree 3rd degree	93(21.8) 249(58.9) 67(15.7) 17(4.0)
Living in the same house	263(61.7)

**Table 2:** Patient characteristics.

Characteristics	n(%)
Age (median/range)	63(19–86)
Diagnosis Gastrointestinal cancer Breast cancer Lung cancer Gynecological cancer Prostate cancer Others	125(29.3) 102(23.9) 61(14.3) 48(11.3) 40(9.4) 50(11.7)
Time to follow-up, months (median/range)	6 (1–274)
Disease status Remission/follow up Under adjuvant therapy Palliativechemotherapy or radiotherapy Palliative care	69(16.2) 79(18.5) 240(56.3) 38(8.9)

The median PTG score was 70.0 (5.0–105.0), and 210 (49.3%) participants had high level PTG scores according to our definition (PTG score ³70.0). In the univariate analysis, being the spouse of the patient, being over 40 years of age, female sex, being married, siblings, and high DAN scores were associated with high PTG scores (Table 3). In multivariate analysis, a high DAN score was the only parameter associated with high PTG scores [OR: 1.6, CI (95%) 1.02–2.5, P = 0.03] (Table 4). The median DAN score was 8.0 (1.0–14.0), and 311 (73%) participants had high level DAN scores according to our definition (DAN score ³7). Caring for elderly patients, female sex, siblings, low income, not working, and a history of psychiatric admission were associated with high DAN scores (Table 3). In multivariate analysis, female sex was the only risk factor for high DAN scores [OR: 1.6, CI (95%) 1.1–2.8, P = 0.049] (Table 4). There was a positive correlation between PTG and DAN scores (r = 0.15, P = 0.001). In addition, higher DAN scores were associated with a positive impact on self-perception (37.0 verus 35.0, P = 0.02), philosophy of life (16.0 versus 13.0, P = 0.035), and changes in relationship (16.0 versus 14.0, P = 0.01). 

**Table 3:** Factors associated with high PTG and DAN scores.

Characteristics	High PTG score	p	High DAN score (n, %)	p
(n, %)	85(49.4) 125(49.2)	0.52	129(75.0) 182(71.7)	0.25
Patient age, years <65 <65	109(47.2) 101(51.8)	0.20	160(69.3) 151(77.4)	0.03
Disease status Remission/follow-up Under adjuvant therapy Palliative chemotherapy or radiotherapy Palliative care	37(53.6) 35(44.3) 115(47.9) 23(60.5)	0.33	49(71.0) 55(69.6) 180(75.0) 27(71.1)	0.76
Degree of relationship Spouse 1st degree 2nd degree 3rd degree Spouse Other	55(59.1) 122(49.0) 26(38.8) 7(41.2) 55(59.1) 155(46.5)	0.07 0.021	73(78.5) 180(72.3) 44(65.7) 14(82.4) 73(78.5) 238(71.5)	0.25 0.11
Living in Same house Another house	127(48.3) 83(50.9)	0.33	189(71.9) 122(74.8)	0.28
Age of the participant <40 <40	95(44.6) 115(54.0)	0.03	159(74.6) 152(71.4)	0.25
Sex Female Male	118(55.1) 92(43.4)	0.01	174(81.3) 137(64.6)	<0.001
Marital status Married Single/divorced	173(53.7) 37(35.6)	0.001	239(74.2) 72(69.2)	0.19
Sibling Present Absent	172(54.4) 38(34.5)	<0.001	240(75.9) 71(64.5)	0.01
Living in City center Town/village	141(49.1) 69(49.6)	0.50	206(71.8) 105(75.5)	0.24
Living in Self contained house Apartment	98(51.3) 112(47.7)	0.25	141(73.8) 170(72.3)	0.40
Monthly income Low (<2000 TL) High (>2000 TL)	116(52.0) 94(46.3)	0.14	172(77.1) 139(68.5)	0.02
Education Illiterate/literate More	17(51.5) 193(49.1)	0.46	27(81.8) 284(72.3)	0.16
Job Retired Working Not working Not working Other	25(50.5) 77(45.0) 108(52.7)	0.33	31(62.0) 113(66.1) 167(81.5) 167(81.5) 144(65.3)	0.001 <0.001
Chronic disease Present Absent	68(49.6) 142(49.1)	0.50	106(77.4) 205(70.9)	0.09
Psychiatry admission Present Absent	39(47.6) 171(49.7)	0.41	66(80.5) 245(71.2)	0.05
DAN score High Low	165(53.1) 45(39.1)	0.007		
PTG score High Low			173(78.6) 138(67.0)	0.005

PTG: Posttraumatic growth, DAN: Death anxiety

**Table 4:** Multivariate analysis of factors associated with high PTG and DAN scores.

	High PTG score				High DAN score			
	Β(SE)	OR	CI (95%)	P	Β(SE)	OR	CI (95%)	P
Being spouse	0.14(0.26)	1.1	0.6–1.9	0.57				
>40 years of age	0.11(0.22)	1.1	0.7–1.7	0.60				
Married	0.47(0.35)	1.6	0.8–3.2	0.11				
Female sex	0.39(0.20)	1.4	0.9–2.2	0.055	0.52(0.27)	1.6	1.1–2.8	0.049
Having sibling	0.28(0.36)	1.3	0.6–2.7	0.43	0.41(0.26)	1.5	0.91– 2.52	0.10
High DAN score	0.48(0.23)	1.6	1.02–2.5	0.03				
Caring elderly patients					0.36(0.23)	1.44	0.9–2.2	0.11
Low income					0.27(0.23)	1.31	0.8–2.0	0.24
Not working					0.45(0.27)	1.58	0.9–2.7	0.10
Chronic disease present					0.001(0.26)	1.001	0.5–1.6	0.99
Psychiatry admission					0.20(0.32)	1.2	0.6–2.3	0.52
High PTG score					0.41(0.23)	1.5	0.9–2.3	0.07

PTG: Posttraumatic growth, DAN: Death anxiety, SE: Standard error

## 4. Discussion

In this study, we tried to look at the positive impacts of a cancer diagnosis on CCPs and planned to analyze the predictors of PTG and DAN. We concluded that high DAN was associated with higher PTG scores, and the female sex was an important factor in death anxiety. We found a statistically significant correlation between PTG and DAN scores.

Trauma has always been a damaging experience, but recent literature concludes that trauma can also lead to positive changes, referred to as posttraumatic growth (PTG) [18]. The PTG model has been defined as follows: some people experience profound changes in their perceptions of themselves, relationships with others, or philosophy of life following their struggle with a major life crisis such as cancer [1]. In addition, PTG has been related to increased self-confidence, the ability to appreciate the present, increased emphasis on family, improved relationships, recognition of new possibilities, and religious growth [19,20]. Although the literature has mostly focused on PTG after the death of cancer patients, a diagnosis of cancer, treatment-related complications, and end of life issues are devastating traumas for CCPs. Female CCPs, older relatives, and those with religious beliefs were reported to have more PTG. Additionally, being the spouse of a cancer patient had positive impacts on spiritual changes [21]. Similar to our results, there are studies in which analysis of sex effects did not yield significant differences [22,23]. In our analysis, participants who were over 40, married, and had siblings were found to have higher PTG, but this was statistically insignificant in multivariate analysis. Balfe et al. studied PTG in caregivers of head and neck cancer patients and showed that increased social support, increasing time since diagnosis, increased worry about cancer, and increased financial stress were associated with more PTG [24]. Ho et al. reported more PTG among those with higher income levels. We could not find any effect of household income and time to follow-up. 

Death anxiety originates from the fear of one’s own death and the dying process. Death anxiety is accepted as an important psychological phenomenon that can damage quality of life [9,10]. Caring for cancer patients may evoke thoughts and fears about personal mortality. Also, CCPs with DAN are prone to increased stress levels, depressive symptoms, and decreased quality of life [25,26]. Female gender and poverty have been associated with higher DAN [10, 27]. In addition, having children, changes in physical appearance, pain, low self-esteem, and physical symptoms have been associated with increased DAN [28] Consistent with the literature, we found that female sex is an important risk factor for increased DAN in CCPs. 

There is limited data addressing whether DAN has a positive impact on our lives. However, as discussed by Irvin D. Yalom, once we confront our mortality, we are inspired to rearrange our priorities, communicate more deeply with those we love, appreciate more keenly the beauty of life, and increase our willingness to take the risks necessary for personal fulfillment. Facing death and overcoming the terror of death can make individuals stare at the sun [29]. Ens et al. reported a positive correlation between DAN and personal growth [30]. There is data supporting the negative effects of worrying about cancer and the fear of recurrence in CCPs [31,32] in terms of psychological morbidity and quality of life. Balfe et al. demonstrated a 7.2-fold increase in the benefit of PTG in CCPs suffering from worry about cancer [24]. Consistent with their data, we demonstrated a 1.6-fold increase in the benefit of PTG in CCPs with DAN. Consistent with our results, Gunst et al. demonstrated a positive impact deriving from fear of death on PTG in adolescent cancer patients [12]. The positive effects of DAN on PTG should be further studied. Religiosity and spirituality are important for coping with the psychological trauma caused by cancer [33]. The data about religious beliefs and DAN is limited. However, religious coping plays an important role for CCPs [10]. Bachner et al. found that religious CCPs experienced more DAN [34]. The association between DAN and PTG should be evaluated based on religiosity and spirituality. 

This study has some inevitable limitations. Firstly, because it is a survey study, there is an unavoidable subjectivity. The population studied in 3 different cancer centers had a heterogeneous socioeconomic background. In addition, we included relatives up to the 3rd degree to evaluate the effects of close relations. However, studying a specific group of relatives can produce more specific results. The CCPs group was young, with a median age of 40. As a result there could be limitations to the analysis of age as the determinant of DAN and PTG. 

 In conclusion; in our study, the female sex was found to be an important risk factor for death anxiety. We found a positive impact of death anxiety on positive psychological changes in CCPs. This is the first indication of the association between DAN and PTG in CCPs. This association should be further studied, including spiritual experiences, religious perspectives, and family relations. 

## Acknowledgements

We would like to thank Hatice Burgaç, Ayşe Arıkanoğlu Soğancı, and Durdu Yaman for their contributions during data collection. No funding was received for this project.

## Informed Consent

The study protocol received institutional review board approval (Çukurova University School of Medicine, Noninterventional Clinical Studies Review Board; date: 13/04/2018, meeting: 76, decision no: 39), and all participants provided informed consent in the format required by the relevant authorities and/or boards.
